# Beam Profiling of Dental Light Curing Units Using Different Camera-Based Systems

**DOI:** 10.1055/s-0041-1731628

**Published:** 2021-08-27

**Authors:** Mateus Garcia Rocha, Dayane Oliveira, Christopher Felix, Jean-François Roulet, Mário Alexandre Coelho Sinhoreti, Américo Bortolazzo Correr

**Affiliations:** 1 Operative Dentistry Division, Department of Restorative Dental Sciences, College of Dentistry, University of Florida, Florida, United States; 2 Bluelight Analytics Inc., Nova Scotia, Canada; 3 Dental Biomaterials Division, Department of Restorative Dentistry, Piracicaba Dental School, State University of Campinas, Sao Paulo, Brazil

**Keywords:** beam profile, light curing units, spectrophotometric analysis, digital cameras

## Abstract

**Objective** This study aimed to perform the beam profile of dental light-curing units (LCUs) using mirrorless and smartphone cameras and correlate it to a camera-based laser beam profiling system.

**Materials and Methods** Three LCUs were evaluated (Radii Plus; Bluephase G2; and VALO Cordless). The spectral power of the LCUs was measured by using a spectrophotometer. The light emitted from the LCUs was projected onto a glass diffuser, and the images were recorded by using a mirrorless camera (NEX-F3), a smartphone (iPhone) and a camera-based beam profiler. Bandpass optical-filters were used, and for each LCU, the total spectral power output was integrated to calibrate the images. Statistical analysis was performed by digital image correlation (pixel by pixel) using Pearson’s correlation (α = 0.05; β = 0.2).

**Results** The beam profile images showed nonuniform radiant emittance and spectral emission distributions across all the LCUs light tip. A strong correlation was found among cameras (Pearson’s r = 0.91 ± 0.03 with 95% confidence interval [CI]: 0.88–0.94 for the NEX-F3 and Pearson’s r = 0.88 ± 0.04 with 95% CI: 0.84–0.92 for the iPhone).

**Conclusion** The standard Ophir beam profile system presented the most accurate distribution, but the mirrorless and smartphone cameras presented a strong correlation in the irradiance distribution of the beam profile images. Alternative cameras can be used to perform light beam profile of dental LCUs, but caution is needed as the type of sensor, image bit depth, and image processing are important to obtain accurate results.

## Introduction

Light curing units (LCUs) are used for light activation of dental adhesives, resin-based composites, resin cements, resin-modified glass ionomers, and many other light-curable dental materials. 1 2 Frequently, manufacturers clearly state that the LCU’s irradiance (mW/cm ^2^ ), and for the most part of the industry, LCUs with an average irradiance higher than 1000 mW/cm are considered decent. 3 It is important to mention that the irradiance is the power (mW) received by a determined area (cm ^2^ ) of a resin-based material, and the radiant emittance (or radiant exitance) is the power (mW) being emitted by a determined area (cm) of the light tip of an LCU. 2 3 Both units (mW/cm ^2^ ) are the quotient of the power (mW) of the light divided by the area (cm ^2^ ), but LCUs with the same radiant emittance (mW/cm ^2^ ) might not have the same irradiance (mW/cm ^2^ ).

However, even if the radiant emittance of an LCU is appropriately described, it does not fully address all significant characteristics of the LCU. Other aspects are extremally important to evaluate the quality of the light emitted, such as the LCU light tip size in comparison to the specimen size, the spectral power, and specially the light beam profile homogeneity. 2 3 4 Thus, to systematically comprehend the emitted-light quality of an LCU, a complete characterization of the light beam emitted by the LCU is essential. 2 3 5

The light beam spectral power distribution of an LCU influences in the polymerization homogeneity of resin-based materials. 6 7 For Monowave LCUs with narrowband spectral emission (within the blue wavelength spectrum only), the use of optical apparatus such as microlenses or optical fiber bundles can collimate the light creating a homogeneous light beam emission. Thus, homogeneously light activating the polymerization of resin-based materials containing only camphorquinone (CQ) as the photoinitiator system. 8 For multiwave LEDs that emit more than one wavelength spectrum (within the violet and blue wavelength spectra), the physical location of the LED chips emitting the different wavelengths seems to be directly correlated with the nonuniform nature of the light beam output of these multiwave LCUs. 9 10 11 Manufacturers’ claim that the combination of multiple LEDs emitting different wavelengths (violet and blue) and different photoinitiators, such as the diphenyl(2,4,6- trimethylbenzoyl)phosphine oxide (TPO) and the benzoyl germanium (Ivocerin), provide better photopolymerization for resin-based materials. 9 However, one of the main obstacles is to build an LCU by using multiple LED chips with a homogeneous light beam emission. 10 11

Therefore, the light beam profile is an important aspect that reveals valuable information about the LCU. A light beam profile can be defined as the power intensity plot of a light beam at a given location along with the light beam output. To characterize the light beam profile, camera-based beam profilers are often used. 12 13 The camera-based beam profiler is an instrument that uses a charge-coupled device (CCD) or complementary metal-oxide-semiconductor (CMOS) camera-based detectors to measure the spatial distribution of light intensity in the cross-section of a light beam according to the conversion of the light photon flux to an electrical current-voltage signal. 14 With the use of bandpass filters, the wavelength distribution of the light beam could also be detected. However, one of the main obstacles of this method is the cost of the complete instrumentation for a camera-based beam profiling system.

The high cost of a beam profiling system is related to the special CCD cameras, which includes a frame grabber card to transcript the signal, a software for controlling the frame grabber card to display beam profiles and to make respective quantitative calculations. Perhaps the most significant reason for this equipment high cost is that those commercially available beam profilers are designed to characterize industrial or medical lasers, and those lasers have an extremely high-powered light beam emission from 10 to 100 W in areas of 1 to 5 mm ^2^ . However, for dental LCU that uses LEDs with power emission from 1 to 5 W in areas of 5 to 10 mm ^2^ , it is not necessary to use equipment with that sophistication.

Thus, it would be great to have low cost equipment that dentists and researchers could get a more robust report of the beam profile of the LCUs that they have been using in their clinics or laboratories. The aim of this study was to demonstrate a method to perform the beam profile of dental LCUs by using low budget cameras and a free open-source software and correlate it to a gold-standard method using camera-based beam profiling system. The research hypothesis was that there are no differences on the beam profiling of different LCUs obtained by using mirrorless, smartphone, and standard camera-based beam profiling systems.

## Materials and Methods

### Spectral Radiant Power

**Fig. 1:**
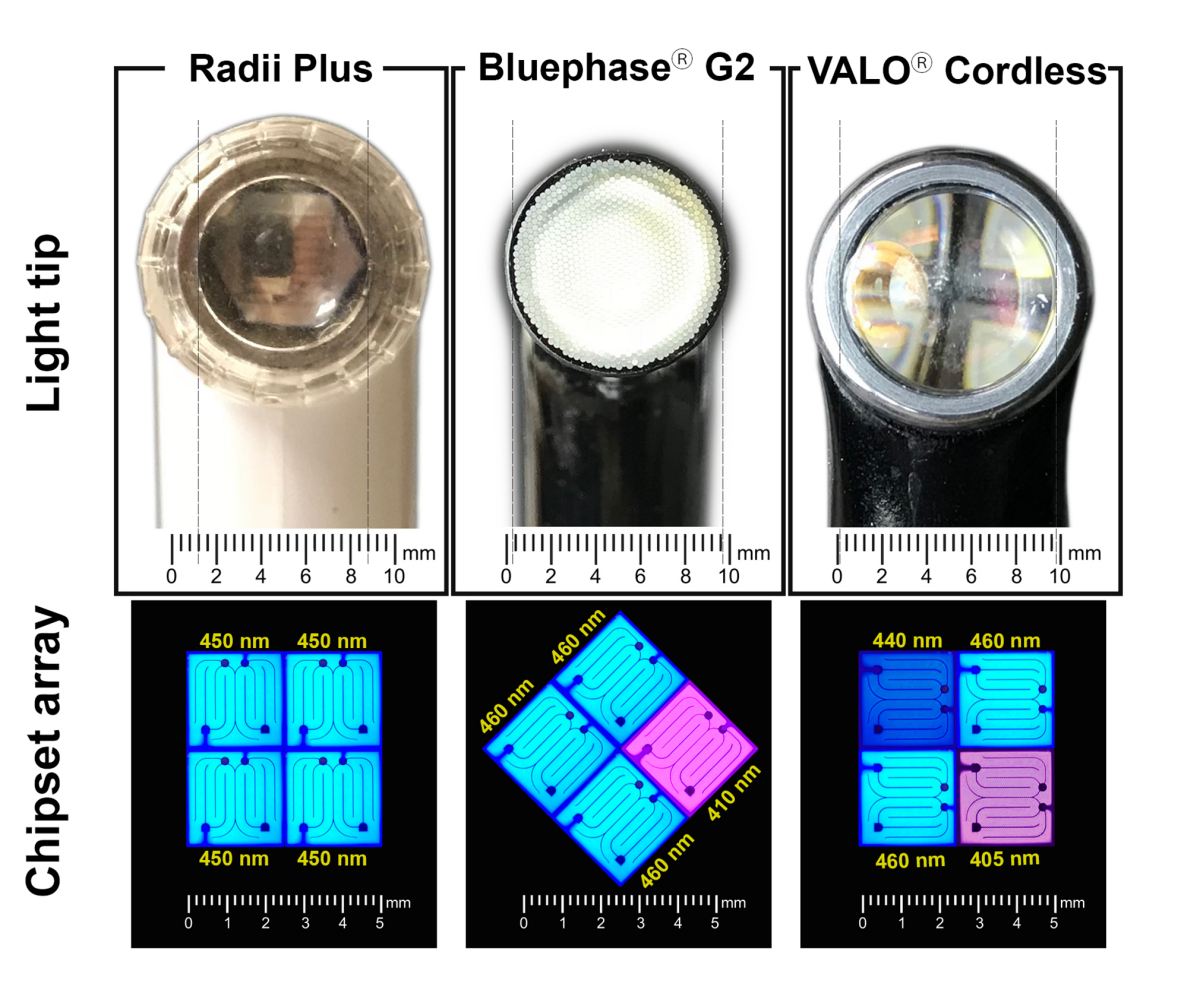
Light tip illustration showing in proportional size the differences between the LCUs ( **[A]** radii plus, **[B]** Bluephase G2, and **[C]** VALO Cordless) and LED chipset array distribution according to the number of LED chips, LED chip wavelength peak of emission, and the position of the LED chips for each LCU. The Chipset Array scheme is based on images collect from the LED chip by using a digital optical microscope (VHX-100, Keyence, Osaka, Japan). LCU, light-curing units

Three LCUs were characterized in this study: Radii Plus (SDI Ltd., Bayswater, Victoria, Australia), Bluephase G2 (Ivoclar Vivadent, Schaan, Liechtenstein), and Valo Cordless (Ultradent Products Inc., South Jordan, Utah, United States). Fig. 1 shows pictures of the light tip and a schematic representation of the chipset array of the LCUs. For each LCU, the area of light emission (cm ^2^ ) was measured by using a digital caliper by the mean of five readings of the inner diameter (d) of the light tip and calculating the area (A _circle_ ) using the formula of the area of a circle (A _circle_ = (d/2)2. The spectral radiant power (mW/nm) of the LCUs was measured five times by using a spectrophotometer with a 16-mm diameter light collection area (MARC Light Collector, BlueLight Analytics, Halifax, Nova Scotia, Canada). 4 For each LCU, the radiant emittance (mW/cm ^2^ ) was calculated by the division of the radiant power (mW/nm) by the area of light emission (cm ^2^ ), and the irradiance (mW/cm ^2^ ) was calculated by the division of the radiant power (mW/nm) by a predetermined circle area of 10 mm in diameter (A = 0.785 cm ^2^ ). The irradiance values in this scenario would represent the exact amount of light delivered in hypothetical situations of light curing a molar tooth with 10 mm of distance mesiodistal or light curing a disk specimen with 10 mm in diameter for in vitro studies.

As specified by the manufacturer, 15 the Radii Plus has four LED chips in the blue wavelength range with an emission peak at 450 nm. The Bluephase G2 has four LED chips, one in the violet wavelength range with a spectral emission peak at 410 nm and the other three in the blue wavelength range with an emission peak at 460 nm. 16 The VALO Cordless light, which also has four LED chips, contains one in the violet wavelength range (spectral peak at 405 nm) and the other three in the blue wavelength range, with one chip emitting at 440 nm and the other two at 460 nm. 17 The radiant emittance (mW/cm ^2^ ) and the irradiance (mW/cm ^2^ ) on the ultraviolet (<380 nm), violet (380–420 nm), royal-blue (420–450 nm), cyan-blue (450–495 nm), green (495–540 nm), as well as, the overall wavelength range (360–540 nm) of each LCU was calculated by integrating the radiant emittance (mW/cm ^2^ ) and the irradiance (mW/cm ^2^ ) versus wavelength curves obtained from the spectrophotometer.

### Beam Profile Image Acquisition

**Fig. 2:**
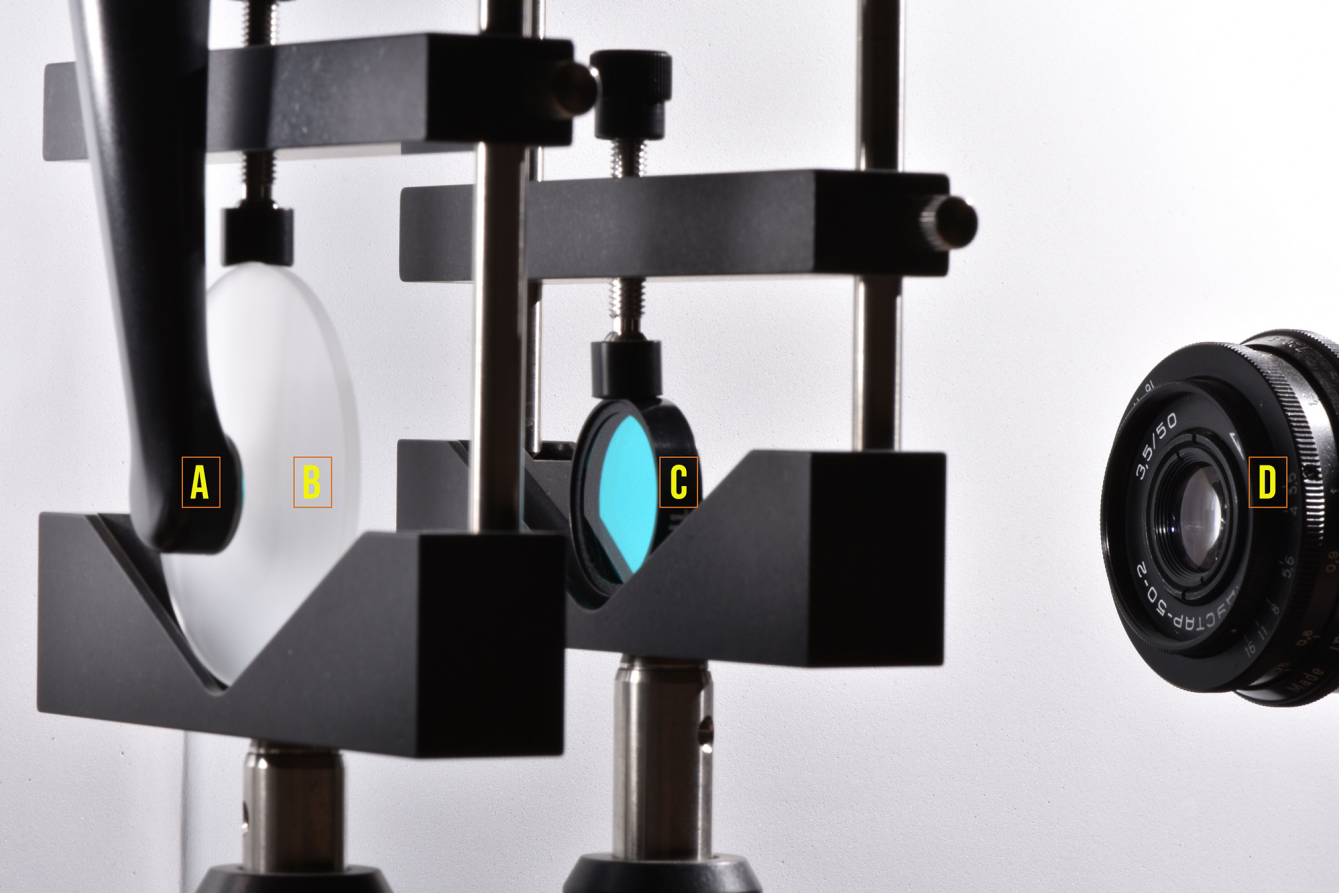
Representative picture of the method setup for the collection of the beam profile data using different cameras: Each LCU ( **A** ) was positioned perpendicular to a glass diffuser ( **B** ) target and pictures were taken by using different cameras ( **D** ) with different bandpass filter ( **C** ) between the glass diffuser and the cameras’ lens; the focal distance between the camera lens and the LCU light tip was 25 cm for the Ophir camera, 28 cm for the NEX-F3 camera, and 10 cm for the iPhone camera. LCU, light-curing units.

A schematic representation of the set up used to make the beam profile images acquisition is showed in Fig. 2 . Each LCU was attached to an x-y-z positioning device mounted on an optical bench (450 × 300 mm breadboard, Edmund Optics, Barrington, New Jersey, United States) to standardize the positioning of the light beam in contact with a diffusive surface of a frosted diffuser glass (DG20–1500, Thorlabs, Inc.). To assess the irradiance distribution according to different wavelength range emission and to narrow the differences in the camera pixel absorption profile, bandpass filters (FB410–10, FB440–10 and FB-460–10, Thorlabs, Inc.) were placed between the diffuser glass and the cameras. As the bandpass filters used in this study has an optical density of approximately 0.2, no neutral density filters were necessary to attenuate the LCU light to avoid pixel intensity saturation on the images. More specific information about the bandpass filter characteristics is provided in the ►Supplementary Material (available in the online version). For the Radii Plus, a bandpass filter centered first at 460 ± 2 nm with a 10 ± 2 nm full width at half maximum (FB460–10, Thorlabs, Inc.) was used to identify the LED chip with a spectrum emission peak at 460 nm. For the Bluephase G2, a bandpass filter centered first at 410 ± 2 nm with a 10 ± 2 nm full width at half maximum (FB410–10, Thorlabs, Inc.) was used to identify the LED chips with a spectrum emission peak at 410 nm. A different bandpass filter, centered at 460 ± 2 nm with a 10 ± 2 nm full width at half maximum (FB460–10, Thorlabs, Inc.) was used to identify the LED chips generating emission peak near 460 nm. For the VALO Cordless, the same bandpass filters described above (FB410–10 and FB460–10) were used to identify the LED chips with spectrum emission peaks at 405 and 460 nm. An additional bandpass filter, centered at 440 ± 2 nm with a 10 ± 2 nm full width at half maximum (FB440–10) was used to identify the LED chip having an emission peak near 440 nm.

The resulting images were recorded by using different cameras: a standard beam profiler CCD camera (Ophir, Model SP503U, Ophir-Spiricon, Logan, Utah, United States), 4 a mirrorless camera (NEX-F3, Sony Corporation, Tokyo, Japan) with 50 mm focal length lens and a smartphone camera (iPhone 7 Plus, Apple Inc., Cupertino, California, United States). The details of the three cameras used in this study (Ophir, NEX-F3 and iPhone) are reported in Table 1 .

**Table 1:** Specification of the cameras used in this study

Camera	Manufacturer	Sensor	Sensor type	Resolution	Sensor Size	Pixel size	Pixel depth (raw format)	Subpixel layout	Dynamic range	Signal-to-noise ratio	Accuracy ^a^
Ophir SP503U	Ophir-Spiricon, Logan, UT, USA	Ophir-Spiricon Silicon CCD	CCD	640 × 480 (0.3 MP)	29.61 mm ^2^ (6.3 × 4.7 mm)	9.90 μm ^2^	12-bit	Monochrome	64 dB	1600:1	0.6 mW
NEX-F3	Sony Corp., Tokyo, Japan	Sony Exmor APS HD - IMX 071	CMOS	4,912 × 3,264 (16 MP)	366.6 mm ^2^ (23.5 × 15.6 mm)	4.78 μm ^2^	14-bit	RGB	49 dB	282:1	3.5 mW
iPhone 7 Plus	Apple Inc., Cupertino, CA, USA	Sony Exmor RS - IMX 315	CMOS	4,032 × 3,024 (12 MP)	32.3 mm ^2^ (5.2 × 6.2 mm)	1.22 μm ^2^	14-bit	RGB	42 dB	125:1	8.0 mW
Abbreviations: CCD, charge-coupled device; CMOS, complementary metal-oxide-semiconductor; RGB, red, green, and blue. ^a^ Considering a light source with 1 W; MP (megapixels); dB (decibel). Data are from technical datasheets of the sensors used in each camera provided by the manufacturers.											

For the Ophir camera, the images were captured by using the BeamGage Standard software (v.6.13.1, Ophir-Spiricon). First, the system was automatically corrected for ambient light and pixel response. Then, the LCUs were powered on and the Ophir SP503U 50 mm lens camera iris was adjusted to use the full dynamic range of the CCD camera without pixel intensity saturation. For the NEX-F3, a sequence of images was captured manually with ISO 200 an aperture of f/32 and shutters speed from 1/4,000 to 1/200. For the iPhone, a series of images was captured manually by using the application software Adobe Photoshop Lightroom CC (v. 3.4.0 F59BE2, Adobe, San Jose, California, United States) to adjust the iPhone 7 Plus rear camera to an ISO 25, an aperture of f/1.8, and a shutter speed from 1/8,000 to 1/200.

The NEX-F3 and the iPhone cameras images were captured in the RAW format with minimally processed data from the image sensor to achieve the maximum dynamic range of both cameras. The images with different times of exposure accordingly to the different shutter speed settings for each camera were examined by histogram analysis of the pixel intensity using the Fiji software to ensure that the images obtained were not saturated (pixel intensity over 65,536 since the images were taken in 16 bit). The images in which the pixel intensity reached the values closer 65,536 and lower than to 65,535 were chosen to be used as the beam profile images.

Each image was individually calibrated according to the power (mW) recorded with the MARC LC for each LCU. The spectral irradiance for violet (peak at 405 nm), royal-blue (peak at 440 nm), and cyan-blue (peak at 460 nm) wavelength regions were recorded and calibrated on separate images with the aid of appropriate bandpass filters. For the Ophir camera, the average power of each LCU was entered into the Beamgage software, and the irradiance distribution was determined by the average pixel intensity distribution interpolated with the size of the area of the light beam. As the Beamgage software offers limited options to customize the graphs and to manipulate the data, the 307,200 calibrated data points from the 640 × 480 matrix array were exported into a scientific graphing and data analysis software (Origin Pro, OriginLab Co., Northampton, Massachusetts, United States).

For the NEX-F3, the images files in the Sony Alpha Raw format (ARW) were transferred to a personal computer, and the images files in ARW were converted into a 16-bit Tagged Image File Format (TIF). The converted images were imported into a free open-source image processing and data analysis software (Fiji, ImageJ, National Institute of Health, Bethesda, Maryland, United States). 18 The same process was performed for the iPhone images. The iPhone images in the Adobe Digital Negative Raw Image file format (DNG) were also converted to a 16-bit TIF file. For NEX-F3 and iPhone cameras, the average power of each LCU was entered into the Fiji software, and the irradiance distribution was determined by the average pixel intensity distribution interpolated with the size of the area of the light beam. Similar to the Beamgage software, the Fiji software offers limited options to customize graphs and manipulate the data. Thus, the calibrated data points were exported into a scientific graphing and data analysis software (Origin Pro, OriginLab Co.).

### Statistical Analysis

Statistical analysis was performed by digital image correlation. Each calibrated beam profile image was transformed into an XYZ matrix array, where the X- and Y-axis represent the location of the pixel and Z represents the calibrated irradiance value (according to the 16-bit grayscale pixel intensity). The data were entered into statistical analysis software (Stata/MP 13, StataCorp, College Station, Texas, United States). For each LCU beam profile image with different wavelength spectrum bandpass filter, the correlation was performed between the control group camera (Ophir) and the mirrorless camera (NEX-F3) and the smartphone camera (iPhone). A correlation analysis was performed by using Pearson’s correlation with α = 0.05 and β = 0.2.

## Results

### Spectral Radiant Power

**Fig. 3:**
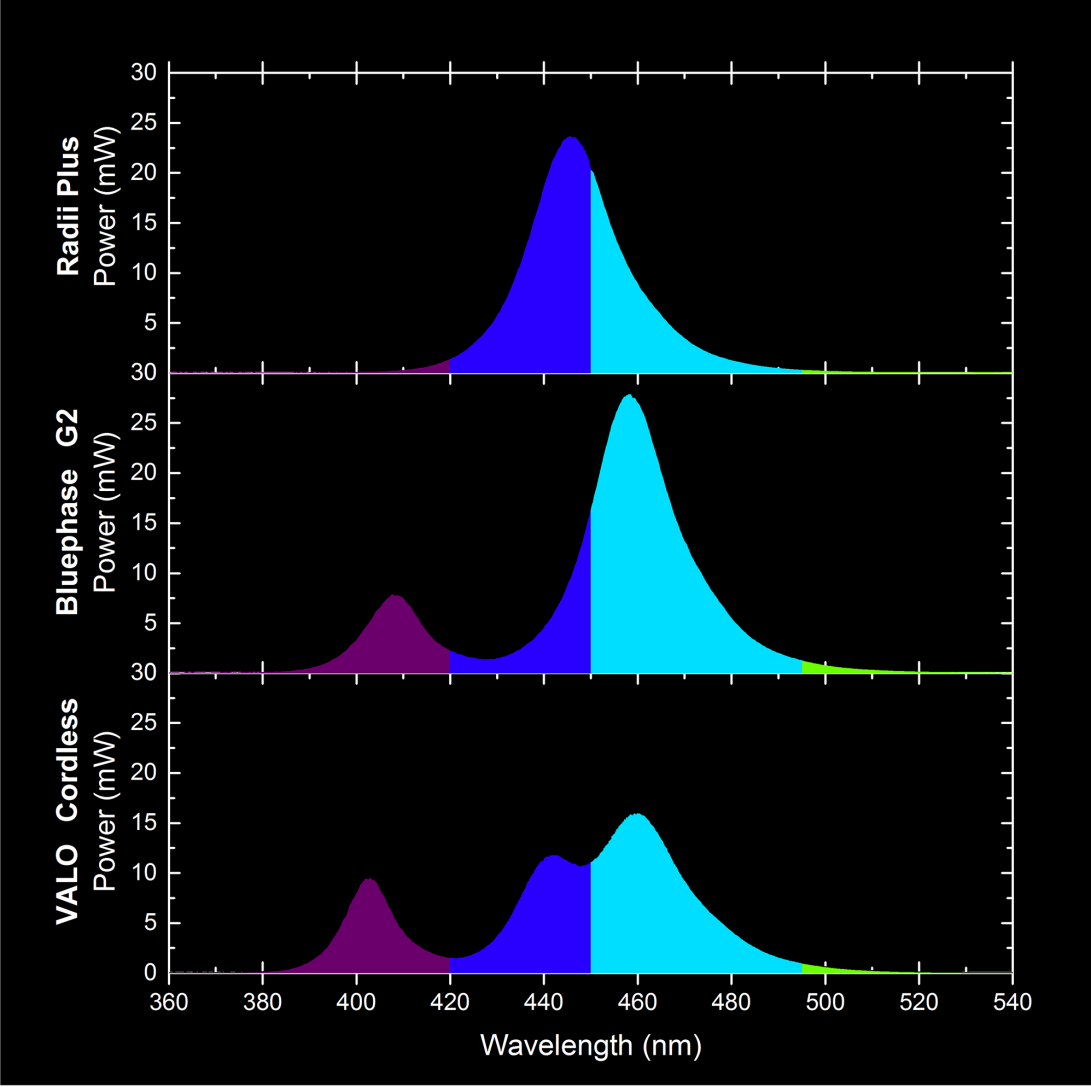
Spectral radiant power (mW/nm) of the Radii Plus, Bluephase G2, and VALO Cordless.

Table 2 shows the total power output (mW), the area of light emission (cm ^2^ ), radiant emittance (mW/cm ^2^ ), and the irradiance (mW/cm ^2^ ) over a 0.765 cm ^2^ area according to the different wavelength ranges. Fig. 3 illustrates the spectral power (mW) distribution according to each wavelength (nm). The Radii Plus had total power of 607 ± 9.3 mW within area of emission of 0.442 cm ^2^ resulting an average radiant emittance of 1,378 ± 8.2 mW/cm ^2^ and an average irradiance of 776 ± 4.6 mW/cm ^2^ . As expected, the majority of the light emission of this monowave LCU was inside “royal-blue” and “cyan-blue” wavelength range with a single peak at 454 nm. For the Bluephase G2 in the high-power mode, the total power was 843 ± 7.8 mW with an area of emission of 0.635 cm ^2^ resulting in an average radiant emittance of 1,305 ± 9.4 mW/cm ^2^ and an average irradiance of 1,056 ± 7.6 mW/cm ^2^ . As the Bluephase G2 is a dual peak multiwave LCU, with one violet LED chip, and three blue LED chips, the light emission of this LCU was inside the “violet,” “royal-blue,” and “cyan-blue” wavelength ranges with peaks at 408 nm (violet) and 458 nm (cyan-blue). For the VALO Cordless in the standard mode, the total power was 843 ± 7.8 mW with an area of emission of 0.635 cm ^2^ resulting in an average radiant emittance of 1,066 ± 2.4 mW/cm ^2^ and an average irradiance of 938 ± 2.1 mW/cm ^2^ . As the VALO Cordless is a triple peak multiwave LCU, with one violet LED chip, one royal-blue LED chip and two blue LED chips, the light emission of this LCU was inside the “violet,” “royal-blue,” and “cyan-blue” wavelength ranges with peaks at 403 nm (violet), 442 nm (royal-blue), and 458 nm (cyan-blue).

**Table 2:** Mean ± standard deviation of the total power output (mW), active area of light emission (cm2), radiant emittance (mW/cm2), and irradiance (mW/cm2) according to the different spectral ranges

Light curing unit		Radii plus	Bluephase G2	VALO cordless
			**(High power mode)**	**(Standard mode)**
Area of light emission (cm ^2^ )		0.442 ± 0.002	0.635 ± 0.001	0.691 ± 0.001
Total (360–540 nm)	Power (mW)	607 ± 9.3	843 ± 7.8	737 ± 9.1
	Radiant Emittance (mW/cm ^2^ )	1,378 ± 8.2	1,305 ± 9.4	1,066 ± 2.4
	Irradiance (mW/cm ^2^ )	776 ± 4.6	1,056 ± 7.6	938 ± 2.1
Ultra-violet (<380 nm)	Power (mW)	0.6 ± 0.1	1 ± 0.5	1 ± 0.6
	Radiant Emittance (mW/cm ^2^ )	1 ± 0.1	2 ± 0.3	2 ± 0.2
	Irradiance (mW/cm ^2^ )	<1	2 ± 0.2	2 ± 0.2
Violet (380–420 nm)	Power (mW)	9 ± 0.2	123 ± 3.9	144 ± 7.1
	Radiant Emittance (mW/cm ^2^ )	81 ± 1.4	190 ± 4.1	210 ± 3.1
	Irradiance (mW/cm ^2^ )	46 ± 0.8	154 ± 3.3	185 ± 2.7
Royal-blue (420–450 nm)	Power (mW)	365 ± 7.6	134 ± 5.2	211 ± 3.3
	Radiant Emittance (mW/cm ^2^ )	1,072 ± 4.0	207 ± 3.2	305 ± 3.1
	Irradiance (mW/cm ^2^ )	604 ± 2.2	167 ± 2.6	268 ± 2.7
Cyan-Blue (450–495 nm)	Power (mW)	230 ± 9.8	572 ± 3.1	369 ± 5.2
	Radiant Emittance (mW/cm ^2^ )	219 ± 4.6	885 ± 4.5	534 ± 6.7
	Irradiance (mW/cm ^2^ )	123 ± 2.6	716 ± 3.6	470 ± 5.9
Blue (420–495 nm) (Royal + Cyan *)*	Power (mW)	595 ± 6.4	706 ± 2.6	580 ± 4.2
	Radiant Emittance (mW/cm ^2^ )	1,346 ± 4.5	1,112 ± 8.1	839 ± 3.6
	Irradiance (mW/cm ^2^ )	778 ± 8.3	923 ± 8.7	758 ± 3.4
Green (495–540 nm)	Power (mW)	3 ± 0.4	13 ± 1.8	11 ± 2.6
	Radiant Emittance (mW/cm ^2^ )	4 ± 0.5	20 ± 1.2	16 ± 0.9
	Irradiance (mW/cm ^2^ )	2 ± 0.3	16 ± 1.0	14 ± 0.8

### Light-Curing Units Beam Profile

**Fig. 4:**
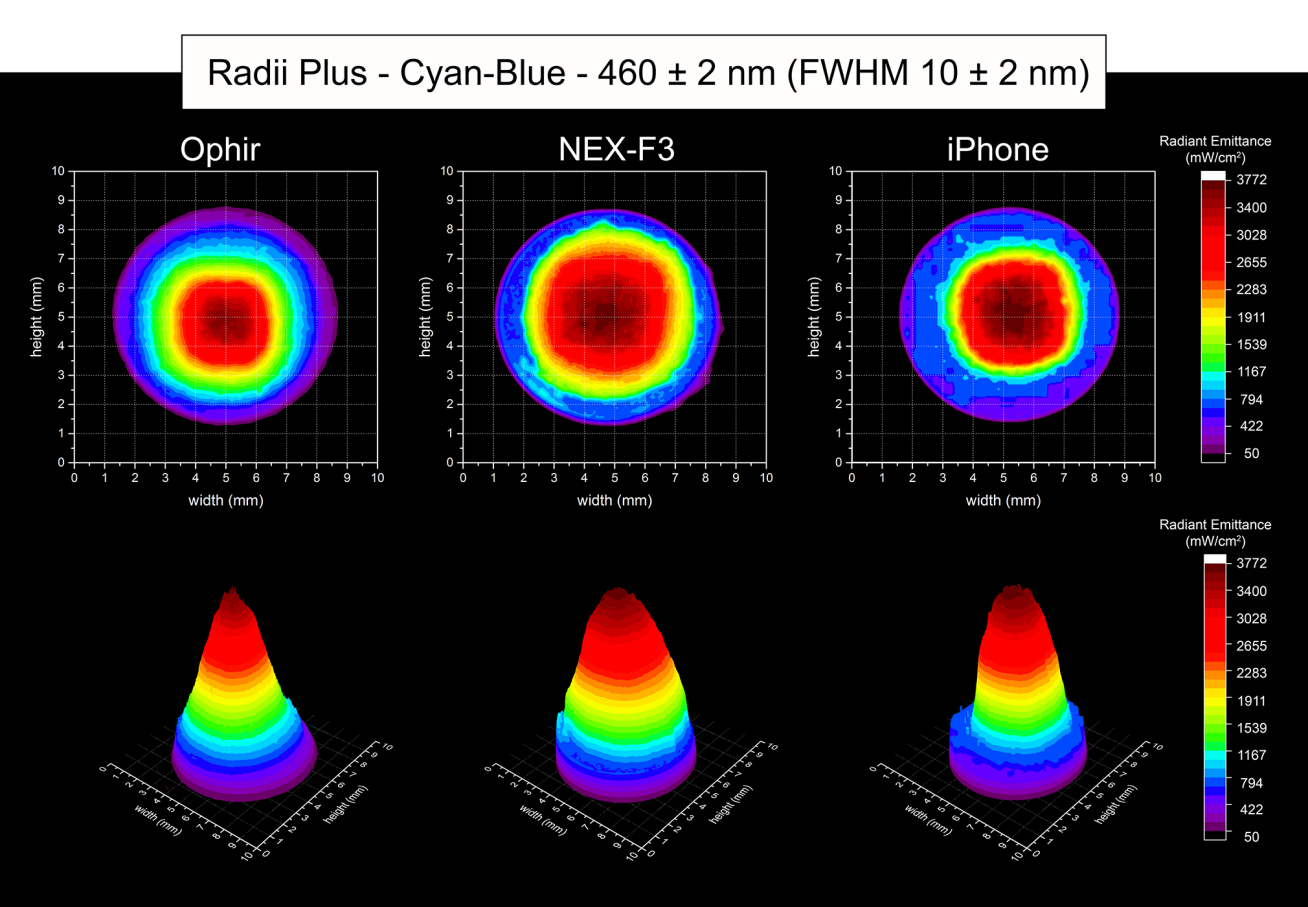
Using the Ophir, NEX-F3 and iPhone cameras: 2D and 3D beam profile images of the Radii plus with the 460 ± 2 nm, full-​width at half-maximum 10 ± 2 nm bandpass filter showing the radiant emittance distribution.

Figs. 4^5^,6,7,8 show the 2D and 3D beam profile images for the different LCUs using the Ophir, NEX-F3, and iPhone cameras. All the images were calibrated accordingly to the radiant emittance (mW/cm ^2^ ) for each respective LCU. Also, the active area of emission (cm ^2^ ), the radiant emittance (mW/cm ^2^ ), and irradiance (mW/cm) distribution of each LCU according to the different wavelength ranges are described in Table 3 .

**Table 3:** Active area of emission (cm2), radiant emittance (mW/cm2) and Irradiance (mW/cm2) distribution of each LCU according to the different wavelength ranges

		Active area of emission (cm ^2^ )	Average		Mode		Minimum		Maximum		Deviation	
			** Radiant Emittance (mW/cm ^2^ ) **	**Irradiance (mW/cm2)**	** Radiant Emittance (mW/cm ^2^ ) **	** Irradiance (mW/cm ^2^ ) **	** Radiant Emittance (mW/cm ^2^ ) **	** Irradiance (mW/cm ^2^ ) **	** Radiant Emittance (mW/cm ^2^ ) **	** Irradiance (mW/cm ^2^ ) **	** Radiant Emittance (mW/cm ^2^ ) **	** Irradiance (mW/cm ^2^ ) **
Radii Plus - Blue 460 ± 2 nm (FWHM 10 ± 2 nm)	Ophir	0.423	1,355	763	385	217	154	86	3,745	2,109	935	527
	NEX-F3	0.425	1,389	782	268	151	186	105	3,466	1,952	1,035	583
	iPhone	0.411	1,298	731	711	400	134	75	3,116	1,754	895	504
Bluephase G2 - Violet 410 ± 2 nm (FWHM 10 ± 2 nm)	Ophir	0.547	195	158	88	71	50	40	584	472	108	87
	NEX-F3	0.545	200	162	150	121	50	40	492	398	112	91
	iPhone	0.608	194	157	178	144	50	40	382	309	71	57
Bluephase G2 - Blue 460 ± 2 nm (FWHM 10 ± 2 nm)	Ophir	0.589	1092	883	933	755	130	105	2,977	2,408	531	430
	NEX-F3	0.604	1,089	881	784	634	109	88	2,253	1,822	538	435
	iPhone	0.603	1,094	885	952	770	112	91	2,350	1,901	595	481
VALO Cordless - Violet 410 ± 2 nm (FWHM 10 ± 2 nm)	Ophir	0.468	209	184	83	73	50	44	555	489	130	114
	NEX-F3	0.433	210	185	103	91	50	44	453	399	104	92
	iPhone	0.465	210	185	84	74	50	44	437	385	113	99
VALO Cordless - Royal-Blue 440 ± 2 nm (FWHM 10 ± 2 nm)	Ophir	0.523	515	453	242	213	106	93	1,075	946	242	213
	NEX-F3	0.608	514	452	692	609	133	117	819	721	133	117
	iPhone	0.574	514	452	248	218	135	119	887	781	208	183
VALO Cordless - Cyan-Blue 460 ± 2 nm (FWHM 10 ± 2 nm)	Ophir	0.609	838	738	1,040	915	117	103	2,106	1,854	430	379
	NEX-F3	0.614	839	739	471	415	120	106	2,115	1,862	433	381
	iPhone	0.613	839	739	372	327	50	44	2,181	1,920	476	419
Note: No statistical differences were found between the different beam profiling systems.													

**Fig. 5:**
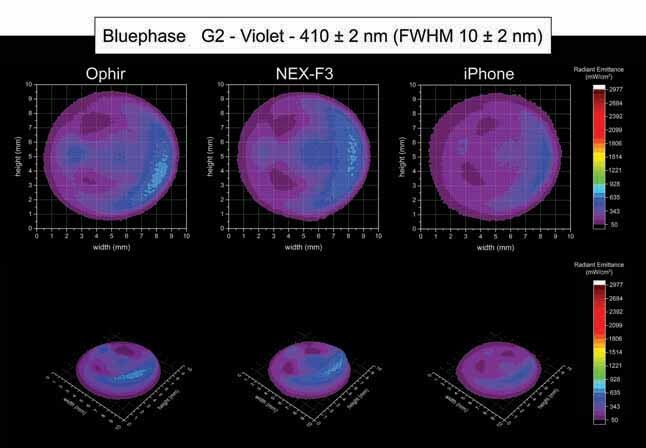
Using the Ophir, NEX-F3 and iPhone cameras: 2D and 3D beam profile images of the Bluephase G2 with the 410 ± 2 nm, full-​width at half-maximum 10 ± 2 nm bandpass filter showing the radiant emittance distribution.

**Fig. 6:**
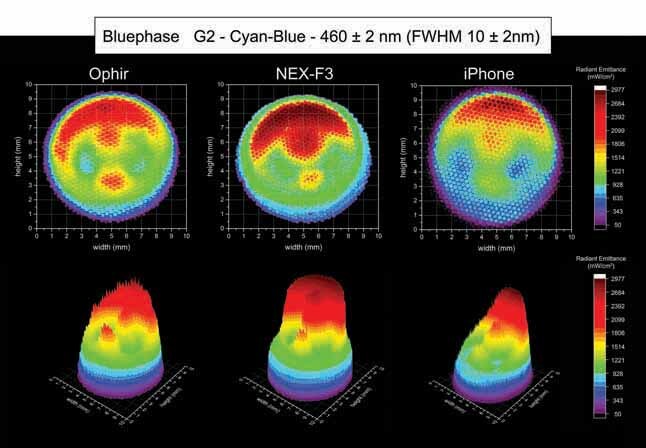
Using the Ophir, NEX-F3 and iPhone cameras: 2D and 3D beam profile images of the Bluephase G2 with the 460 ± 2 nm, full-​width at half-maximum 10 ± 2 nm bandpass filter showing the radiant emittance distribution.

**Fig. 7:**
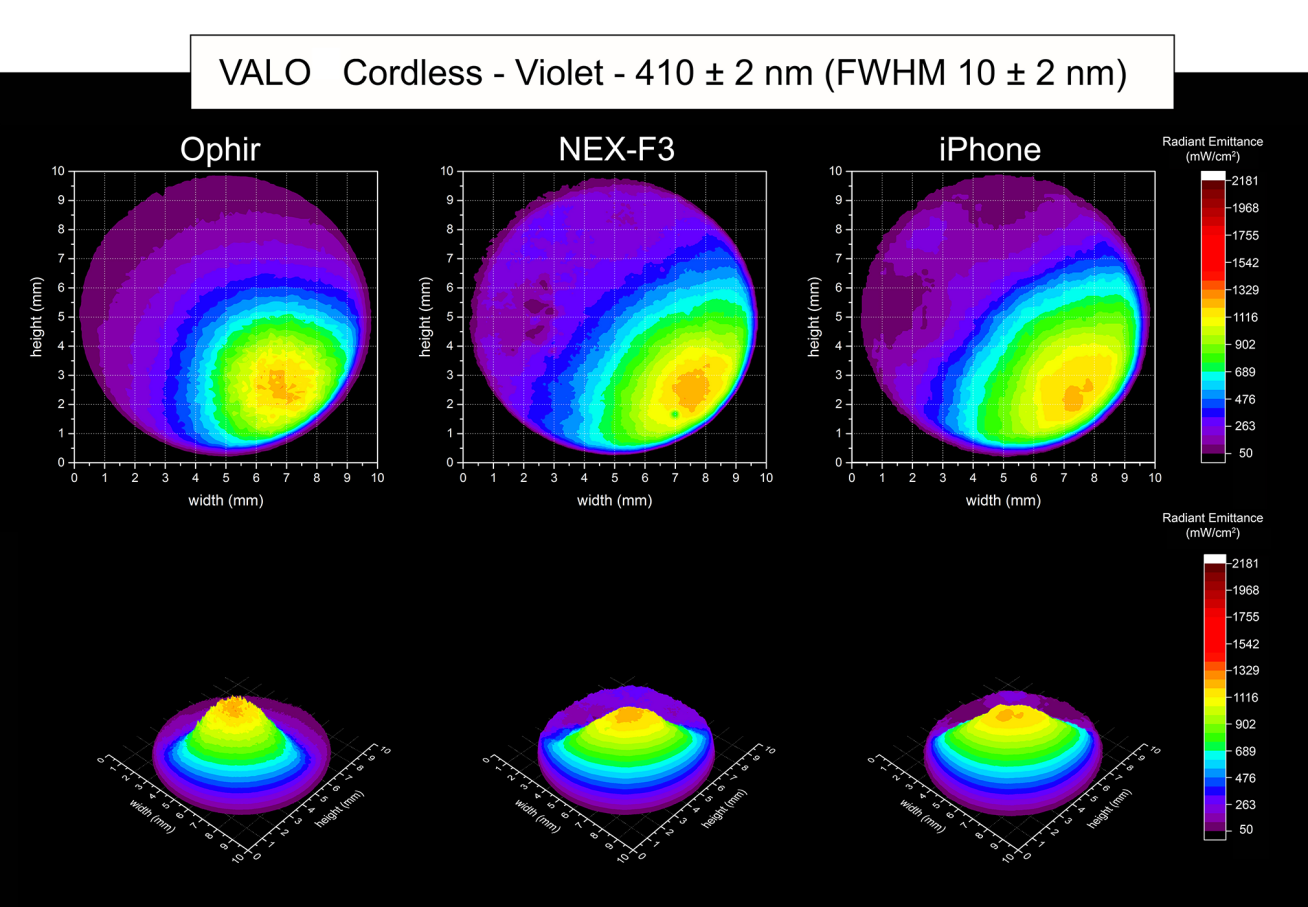
Using the Ophir, NEX-F3 and iPhone cameras: 2D and 3D beam profile images of the VALO Cordless with the 410 ± 2 nm, full-​width at half-maximum 10 ± 2 nm bandpass filter showing the radiant emittance distribution.

**Fig. 8:**
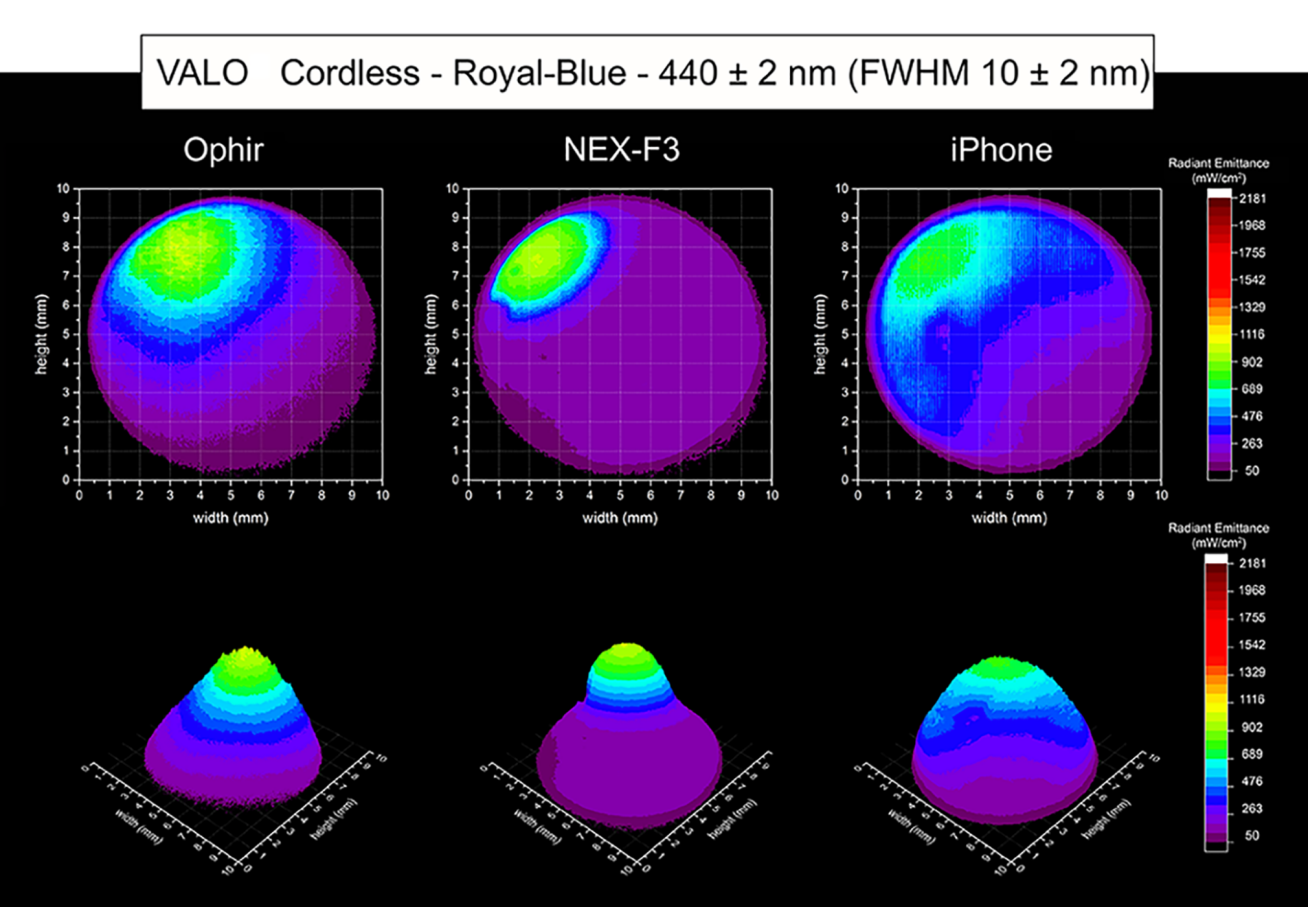
Using the Ophir, NEX-F3 and iPhone cameras: 2D and 3D beam profile images of the VALO Cordless with the 440 ± 2 nm, full-​width at half-maximum 10 ± 2 nm bandpass filter showing the radiant emittance distribution.

Fig. 4 shows the 2D and 3D beam profile images for the Radii Plus using the Ophir, NEX-F3, and iPhone cameras. The Radii Plus had a higher light emission at the center part of the light tip and a lower radiant emittance on the boundaries of the light tip emission. Using the Ophir camera for the Radii Plus beam profile analysis, it was detected an average radiant emittance of 1,355 mW/cm ^2^ , with a maximum radiant emittance of 3,745 mW/cm ^2^ and a minimum radiant emittance of 154 mW/cm ^2^ , inside an active area of emission of 0.423 cm ( Table 3 ). Using the NEX-F3 camera for the Radii Plus beam profile analysis, it was detected an average radiant emittance of 1,389 mW/cm ^2^ , with a maximum radiant emittance of 3,466 mW/cm ^2^ and a minimum radiant emittance of 186 mW/cm ^2^ , inside an active area of emission of 0.425 cm ^2^ . Using the iPhone camera for the Radii Plus beam profile analysis, it was detected an average radiant emittance of 1,298 mW/cm ^2^ , with a maximum radiant emittance of 3,116 mW/cm ^2^ and a minimum radiant emittance of 134 mW/cm ^2^ , inside an active area of emission of 0.411 cm ^2^ .

Figs. 5 and 6 show the 2D and 3D beam profile images for the Bluephase G2 using the Ophir, NEX-F3, and iPhone cameras within the violet spectrum and the blue spectrum, respectively. For the Bluephase G2, an area with higher light emission was detected on the boundaries of the light tip. However, according to the LED chips distribution, the violet and blue spectrum were placed in different regions across the light tip. Within the violet spectrum (410 ± 2 nm, FWHM 10 ± 2 nm), an area of higher radiant emittance was detected on the left side of the light tip, where the violet LED chip is located inside the body of the LCU next to an optical reflector. Using the Ophir camera for the analysis of the Bluephase G2 inside the violet spectrum (410 ± 2 nm, FWHM 10 ± 2 nm), an average radiant emittance of 195 mW/cm ^2^ was detected, with a maximum radiant emittance of 584 mW/cm ^2^ and a minimum radiant emittance of 50 mW/cm ^2^ , inside an active area of emission of 0.547 cm ( Table 3 ). When the NEX-F3 camera was used for the beam profile analysis of the Bluephase G2 inside the violet spectrum (410 ± 2 nm, FWHM 10 ± 2 nm), an average radiant emittance of 200 mW/cm ^2^ was detected, with a maximum radiant emittance of 492 mW/cm ^2^ and a minimum radiant emittance of 50 mW/cm ^2^ , inside an active area of emission of 0.545 cm ^2^ . For the iPhone camera, the Bluephase G2 inside the violet spectrum (410 ± 2 nm, FWHM 10 ± 2 nm) beam profile analysis detected an average radiant emittance of 194 mW/cm ^2^ , with a maximum radiant emittance of 382 mW/cm ^2^ and a minimum radiant emittance of 50 mW/cm ^2^ , inside an active area of emission of 0.608 cm ^2^ .

Considering the beam profile analysis of the Bluephase G2 within the blue spectrum (460 ± 2 nm, FWHM 10 ± 2 nm), an area of higher radiant emittance on the top side of the light tip was detected, where one of the LED chips emitting blue light is located inside the body of the LCU next to an optical reflector. The Ophir camera for the Bluephase G2 inside the blue spectrum (460 ± 2 nm, FWHM 10 ± 2 nm) beam profile analysis detected an average radiant emittance of 1,092 mW/cm ^2^ , with a maximum radiant emittance of 2,977 mW/cm ^2^ and a minimum radiant emittance of 130 mW/cm ^2^ , inside an active area of emission of 0.589 cm ( Table 3 ). The NEX-F3 camera for the Bluephase G2 inside the blue spectrum (460 ± 2 nm, FWHM 10 ± 2 nm) beam profile analysis detected an average radiant emittance of 1089 mW/cm ^2^ , with a maximum radiant emittance of 2,253 mW/cm ^2^ and a minimum radiant emittance of 109 mW/cm ^2^ , inside an active area of emission of 0.604 cm ^2^ . The iPhone camera for the Bluephase G2 inside the blue spectrum (460 ± 2 nm, FWHM 10 ± 2 nm) beam profile analysis detected an average radiant emittance of 1,094 mW/cm ^2^ , with a maximum radiant emittance of 2,350 mW/cm ^2^ and a minimum radiant emittance of 112 mW/cm ^2^ , inside an active area of emission of 0.603 cm ^2^ .

Figs. 7^8^,9 show the 2D and 3D beam profile images for the VALO Cordless using the Ophir, NEX-F3, and iPhone cameras within the violet spectrum, the royal-blue spectrum and cyan-blue spectrum), respectively. The VALO Cordless had a higher light emission on the boundaries of the light tip emission. However, according to the distribution of the LED chips, the violet, the royal-blue, and the cyan-royal blue spectra were located in different regions of the light tip.

**Fig. 9:**
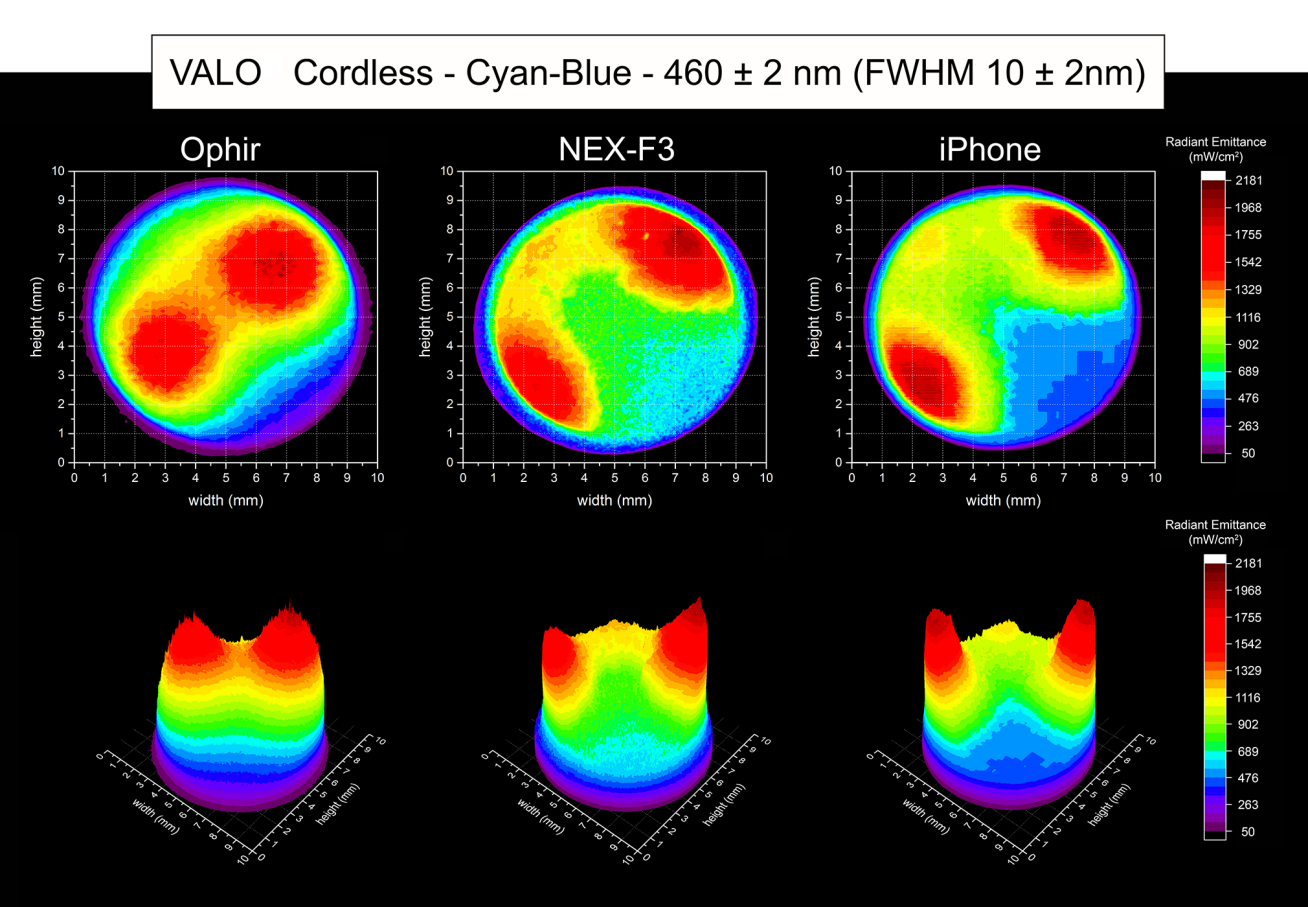
Using the Ophir, NEX-F3 and iPhone cameras: 2D and 3D beam profile images of the VALO Cordless with the 460 ± 2 nm, full-​width at half-maximum 10 ± 2 nm bandpass filter showing the radiant emittance distribution.

Within the violet spectrum (410 ± 2 nm, FWHM 10 ± 2 nm), the VALO Cordless had an area of higher radiant emittance on the right-bottom side of the light tip, where the violet LED chip is located inside the head of the LCU. The Ophir camera for the VALO Cordless inside the violet spectrum (410 ± 2 nm, FWHM 10 ± 2 nm) beam profile analysis detected an average radiant emittance of 209 mW/cm ^2^ , with a maximum radiant emittance of 555 mW/cm ^2^ and a minimum radiant emittance of 50 mW/cm ^2^ , inside an active area of emission of 0.468 cm ( Table 3 ). The NEX-F3 camera for the VALO Cordless inside the violet spectrum (410 ± 2 nm, FWHM 10 ± 2 nm) beam profile analysis detected an average radiant emittance of 210 mW/cm ^2^ , with a maximum radiant emittance of 453 mW/cm ^2^ and a minimum radiant emittance of 50 mW/cm ^2^ , inside an active area of emission of 0.433 cm ^2^ . The iPhone camera for the VALO Cordless inside the violet spectrum (410 ± 2 nm, FWHM 10 ± 2 nm) beam profile analysis detected an average radiant emittance of 210 mW/cm ^2^ , with a maximum radiant emittance of 437 mW/cm ^2^ and a minimum radiant emittance of 50 mW/cm ^2^ , inside an active area of emission of 0.465 cm ^2^ .

Inside the royal-blue spectrum (440 ± 2 nm, FWHM 10 ± 2 nm), the VALO Cordless had an area of higher radiant emittance on the top and right side of the light tip, where one of the royal-blue LED chip is located inside the body of the LCU. The Ophir camera for the VALO Cordless inside the blue spectrum (440 ± 2 nm, FWHM 10 ± 2 nm) beam profile analysis detected an average radiant emittance of 515 mW/cm ^2^ , with a maximum radiant emittance of 1,075 mW/cm ^2^ and a minimum radiant emittance of 106 mW/cm ^2^ inside an active area of emission of 0.523 cm ^2^ . The NEX-F3 camera for the VALO Cordless inside the royal-blue spectrum (440 ± 2 nm, FWHM 10 ± 2 nm) beam profile analysis detected an average radiant emittance of 514 mW/cm ^2^ , with a maximum radiant emittance of 819 mW/cm ^2^ and a minimum radiant emittance of 133 mW/cm ^2^ , inside an active area of emission of 0.608 cm ^2^ . The iPhone camera for the VALO Cordless inside the royal-blue spectrum (440 ± 2 nm, FWHM 10 ± 2 nm) beam profile analysis detected an average radiant emittance of 514 mW/cm ^2^ , with a maximum radiant emittance of 887 mW/cm ^2^ and a minimum radiant emittance of 135 mW/cm ^2^ , inside an active area of emission of 0.574 cm ^2^ .

Within the cyan-blue spectrum, the VALO Cordless had an area of higher radiant emittance on the top-right and bottom left side of the light tip, where each of two blue LED chips are located inside the head of the LCU. The Ophir camera for the VALO Cordless inside the cyan-blue spectrum (460 ± 2 nm, FWHM 10 ± 2 nm) beam profile analysis detected an average radiant emittance of 838 mW/cm ^2^ , with a maximum radiant emittance of 2,106 mW/cm ^2^ and a minimum irradiance of 117 mW/cm ^2^ , inside an active area of emission of 0.609 cm ( Table 3 ). The NEX-F3 camera for the VALO Cordless inside the cyan-blue spectrum (460 ± 2 nm, FWHM 10 ± 2 nm) beam profile analysis detected an average radiant emittance of 839 mW/cm ^2^ , with a maximum radiant emittance of 2,115 mW/cm ^2^ and a minimum radiant emittance of 120 mW/cm ^2^ , inside an active area of emission of 0.614 cm ^2^ . The iPhone camera for the VALO Cordless inside the cyan-blue spectrum (460 ± 2 nm, FWHM 10 ± 2 nm) beam profile analysis detected an average radiant emittance of 839 mW/cm ^2^ , with a maximum irradiance of 2,181 mW/cm ^2^ and a minimum radiant emittance of 50 mW/cm ^2^ , inside an active area of emission of 0.613 cm ^2^ .

Table 4 shows the Pearson’s correlation of the images pixel intensities according to the x and y position using the NEX-F3 and iPhone cameras in comparison to the Ophir camera. A strong correlation in the pixel intensity distribution was found between the Ophir camera and the NEX-F3 camera (Pearson’s r = 0.91 ± 0.03 with 95% CI: 0.88–0.94) and as well between the Ophir camera and the iPhone camera (Pearson’s r = 0.88 ± 0.04 with 95% CI: 0.84–0.92 for the iPhone).

**Table 4:** Correlation of the images using the NEX-F3 and iPhone cameras versus the Ophir camera

Light curing unit	Wavelength	NEX-F3			iPhone		
		**Pearson’s r**	**Adjusted R-square**	***p*** **-Value**	**Pearson’s r**	**Adjusted R-square**	***p*** **-Value**
Radii Plus	Blue	0.92	0.85	0.0001	0.89	0.79	0.0001
Bluephase G2	Violet	0.94	0.89	0.0001	0.88	0.78	0.0001
	Blue	0.92	0.84	0.0001	0.85	0.72	0.0001
VALO Cordless	Violet	0.93	0.87	0.0001	0.95	0.89	0.0001
	Royal-Blue	0.86	0.74	0.0001	0.84	0.71	0.0001
	Cyan-Blue	0.89	0.79	0.0001	0.86	0.73	0.0001

## Discussion

The research hypothesis that the mirrorless and smartphone cameras would have the same performance as the camera-based beam profiling systems was accepted. As mentioned in this study introduction, the Ophir camera-based laser beam profiling system and other elaborated beam profile systems are considered the best quality scientific equipment regarding the characterization of light beams of medical and industrial laser. Those beam profiling systems are proven to be also reliable to characterize the light beam profile of dental LCUs. 1 2 7 However, in terms of characterizing a dental LCU, the use of more accessible technologies can provide results with similar quality but at low cost. Nevertheless, it is important to state that there are some differences between the Ophir camera and the NEX-F3 and the iPhone cameras used in this study. Indeed, these differences found in the NEX-F3 camera (mirrorless) and in the iPhone (Smartphone) camera might be the same differences observed in other models of mirrorless and smartphone cameras, since these electronical devices share similar camera technology. 19

The first difference is that the Ophir camera uses a charge couple devices (CCD) sensor while the NEX-F3 and the iPhone cameras use a complementary metal-oxide semiconductor (CMOS) sensor. The CCD and CMOS sensors operate quite similar using an array of photodetectors. Then, when a photon from the LCU reaches the photodetector, a photoelectric conversion occurs generating electrons in each photodetector. 20 21 22 The number of electrons generated in each photodetector is proportional to the number of photons that have reached the sensor. However, the difference between CCD and CMOS sensors is in the conversion of the analog electron signal in each photodetector to a digital signal in a form of image pixel. 20 Basically, for the CMOS sensor, each photodetector has its motherboard processor located at each pixel, which converts the analog to the digital signal. On the other hand, the motherboard processor of a CCD sensor that converts the analog to the digital signal is located nearest to the edge of the sensor. 21 Thus, the photodetectors on the edge of the sensor transfer their electrons to the motherboard processor. However, all other photodetectors transfer their electron charge to the neighboring photodetector that is closer to the motherboard and the process continues when these photodetectors transfer the electrons to the motherboard processor. 20 21 22 Once the motherboard processes the analog signal and transforms it into a digital signal, this process continues until all electrons captured in an image are detected and converted into digital. In this way, the electrons are removed from the sensor one photodetector at a time. 20 The fact is that for many years, CCD sensor was the predominant sensor used in laser beam profiling applications. Also, one of the foremost reasons behind the CCD use was their lower noise level, which means that the images are smoother and the pixel intensity values are more accurate.

However, recent advances in CMOS technology have steadily reduced the CMOS noise. As shown in Table 1 , the dynamic range of the Ophir camera (64 dB) is higher than the NEX-F3 (49 dB) and iPhone (42 dB) cameras. The dynamic range is the range that a sensor can detect from the lowest light intensity to the higher light intensity. That means that the Ophir camera can detect differences in a wider range than the NEX-F3 and iPhone camera. It is known that a camera with a higher dynamic range would have a lower signal-to-noise ratio and, as a consequence, a higher accuracy to detected differences in the power of the light beam. 19 23 24 When considering the dynamic range of each camera in a minimum detection threshold of 50 mW/cm ^2^ for the characterization of a dental LCU, the Ophir camera can detect differences without pixel saturation up to 79,244.5 mW/cm ^2^ with an accuracy of 0.6 mW/cm ^2^ . While, the NEX-F3 can detect differences without pixel saturation up to 14,091.9 mW/cm ^2^ with an accuracy of 3.5 mW/cm ^2^ , and the iPhone can detect differences without pixel saturation up to 6,294.6 mW/cm ^2^ , with an accuracy of 3.5 mW/cm. Indeed, the Ophir camera is by far better than the NEX-F3 and iPhone cameras. However, a recent study 4 has shown that the maximum irradiance of the majority dental LCUs might have a range from 1,508 to 5,950 mW/cm ^2^ , which means that both NEX-F3 and iPhone are still capable of performing the light collection without sensor oversaturation. However, some exceptions would be expected, such as the use of the iPhone camera for the beam profile characterization of the S.P.E.C. 3 (Coltene/Whaledent AG, Altstätten, Switzerland) that had a maximum irradiance of 8,325 mW/cm. 4 Thus, when choosing a low-budget camera to do a beam profile of a dental LCU, look for a CCD or a CMOS camera that has a dynamic range higher than 41 dB.

Regarding the image resolution, the NEX-F3 and iPhone cameras have better resolution with more megapixels and smaller pixel sizes, which means that in terms of spatial distribution, those cameras would have better precision than the Ophir camera. However, these results do not rule out the influence of other factors such as the subpixel layout, as shown in Table 1 . The subpixel layout defines if a silicon-based camera sensor can detect light signals in grayscale or color by means of pixel intensities in different color channels (red, green, and blue [RGB]). In general, all camera sensors are currently monochrome, the photodiodes sensors that accept the photons and convert them into electrons, and so forth, all of them only deliver a level of gray as the sort of quantitative pixel intensity information. 21 However, if filters are placed in front of them, levels of gray of a blue, green, or red channels can be detected. The mirrorless and smartphone cameras are designed to capture images with color depths; thus, necessarily, a microfilter in a Bayer array pattern with RGB in a 1:2:1 proportion is placed in front of the sensors. 21 Considering that the wavelength range of the dental LCUs is within the blue channel of the Bayer array, it is almost certain that three quarters of the total pixels captured by the sensor are lost. As an example, the images obtained with the NEX-F3 and iPhone had higher resolution and more megapixels than the Ophir camera. However, it does not mean that after transforming the color image (color-chrome) obtained using the NEX-F3 and iPhone into a black and white image (monochrome), the transformed monochrome images would have better resolution than the original monochrome images obtained using the Ophir camera. The fact is that when the color-chrome images are transformed into a monochrome image, the color-coded pixels (red, green, or blue) are interpolated and reduced by four. This interpolation reduces the image resolution by estimating the pixel intensity from surrounding pixel intensity known values. As a consequence, making the image acquisition with larger pixels and more noise. Thus, it is suggested that when using color-chrome mirrorless or smartphone cameras for beam profile purposes that the camera should have at least 2,560 × 1,920 pixels (~5.0 megapixel) of resolution to get similar results as the standard Ophir cameras.

When a photon from a dental LCU reaches the detector of a beam profile camera, its energy is converted from an electron-volt analog signal into a digital signal, as fully explained in many previous studies. 22 Also, as mentioned before, the dynamic range is the range that a sensor can detect from the lowest light intensity to the highest light intensity. However, from the lowest to the highest pixel value, there is a continuous variable that could have an infinite number of possible values. Thus, the relation between the lowest to the highest signal detected by the sensor, associated with the amount of data stored digitally, defines the bit depth of the beam profile images. 23 The bit depth is the number of intervals, or steps, between the minimum and the maximum pixel value, which is essential to differentiate the threshold between the value of two different pixels (the pixel value, for the beam profile images represents the irradiance (mW/cm ^2^ )). Digital cameras usually have 8-bit (2 ^8^ = 256), 10-bit (2 ^10^ = 1,024), 12-bit (2 ^12^ = 4,096), or 16-bit (2= 65,536). 19 In terms of beam profile analysis, if a camera sensor has 8-bit, there is 256 levels for the pixel value; this means that if a dental LCU with a maximum irradiance of 1,000 mW/cm ^2^ is being evaluated, the camera can detect differences in irradiance of 3.9 mW/cm. Still, it is important to mention that if images are captured in JPEG format, the bit depth is limited to 8-bit, which gives 256 levels of gray and as consequence, 256 levels of irradiance, regardless of the bit depth of your camera sensor. 24 However, images captured in RAW/DNG format can be anywhere from 10- to 16-bit, with the latter giving 65,536 levels of grays, meaning that it has a lot more accuracy in detect differences between pixels and when those images are converted to a TIFF file, there is no compression or loose of data. Thus, for the use of mirrorless or smartphone cameras as a beam profiling analysis system, the camera RAW file should be accessible because besides shooting the images without any digital processing, the images have more information (bit depth) about the dental LCU analyzed.

One of the main obstacles in beam profile analysis is to calibrate the images properly. The reason that occurs is because the absolute power fed to the camera is relative to the total power of the light beam. 13 22 Therefore, it is essential to have an accurate method to collect the total power of the dental LCU. Otherwise, the irradiance distribution of the beam profile images would not be consistent. Several methods have been reported in the literature to collect the power (mW) and the spectral irradiance (mW/cm/nm) of LCUs. 4 7 12 13 It is almost certain that the method that uses an integrated sphere coupled to a spectrometer is the most reliable and precise of all methods. 4 However, alternatives to that are also valid, but a note of caution is due here because each of these methods has their limitations. 25 26 In this study, a portable-spectrometer with a 16-mm in diameter area of the collection was used to collect the spectral power (mW/nm) and the spectral irradiance (mW/cm/nm) of the dental LCUs from the entire light tip. It seems possible that other methods using a spectrometer coupled with a cosine corrector (MARC Resin Calibrator, BlueLight Analytics) might work as well, but it is important to mention that the power collected should be calibrated accordingly to the size of the cosine corrector (usually 3.9 mm in diameter) and not accordingly to the size of the light tip. In addition, cameras do not have uniform wavelength absorption. 27 Therefore, they would have a different calibration factor for every wavelength of the light source that is used, and the attempt to calibrate the camera as a function of wavelength by using bandpass filters is the best way to get the best correlation among cameras. 27 In this study, the intended purpose was to separate the royal- and cyan-blue spectrum, so a bandpass filter with a FWHM 10 ± 2 nm was used. However, for more information regarding the use of bandpass filters with FWHM of 40 nm that covers the royal- and cyan-blue spectrum together, refer to the supporting information provided in this article.

There is a strong correlation between the dental LCU beam profile, and the physical and chemical properties of resin-based materials have been reported in the literature. 6 7 28 29 The results of the present study broadly support the work of other studies that the beam profile of the dental LCU are notably different. 4 7 There are many LCUs available in the market with a considerable variation in the number of LED chips, light tip sizes, and so many other aspects. All these variations can affect the beam profile of dental LCU, and it is essential to measure the light beam profile in any application if the energy distribution affects the performance of the dental LCU for its intended purpose.

The beam profile characterization of the Radii Plus, which represents a monowave LED LCU, showed big discrepancies between the radiant emittance at the center of the light tip and the irradiance in areas on the boundary of the light tip, regardless the camera used for beam profile analysis. The Radii Plus has a light tip with an external dimension of 1.2 cm in diameter. However, the inner exit window of the microlens has only 0.75 cm in diameter, resulting in an area of emission 0.442 cm ^2^ . Thus, Radii Plus had an average radiant emittance of approximately 1,378 mW/cm ^2^ . But there were areas of higher radiant emittance at the center of the light tip with approximately 3,700 mW/cm ^2^ , and areas of low radiant emittance at the periphery of the light tip with 160 mW/cm ^2^ . One possible reason for these findings is probably due to the Radii Plus LCU design. The Radii Plus consist of four high-power LED chips at the center of the light tip associated to a plastic microlens that does not have much optical capability of mixing and collimate the light beam throughout the light tip.

The Bluephase G2 is a multiwave LED LCU with two peaks of emission. The beam profile showed differences in the radiant emittance and in the spectral radiant emittance regardless of the camera used for the analysis. The Bluephase G2 has a fiber optic light tip with an incoherent bundle and an external dimension of 1.0 cm in diameter. However, the active area of emission has 0.92 cm in diameter, resulting in an area of emission 0.635 cm ^2^ . The Bluephase G2 had an average radiant emittance of approximately 1,305 mW/cm ^2^ . However, for the violet wavelength range, there were areas of higher radiant emittance (~555 mW/cm ^2^ ) at the left part of the light tip and areas of low radiant emittance (~50 mW/cm ^2^ ) on the bottom left of the light tip. For the blue wavelength range, there were areas of higher radiant emittance (~3,000 mW/cm ^2^ ) at the top part of the light tip, and areas of low radiant emittance (~130 mW/cm ^2^ ) on the bottom left of the light tip. These findings are also probably related to the Bluephase G2 design because the Bluephase G2 has four high-power LED chips emitting different wavelengths, three LED chips emitting blue light, and one LED chip emitting violet light). All four LED chips are located at the center of the body of the LCU associated with diffusive reflectors that orientate the light beam toward the boundary of the light tip. Plus, the coherent light tip bundle from the Bluephase G2 does not have any optical capability of mixing and collimate the light beam throughout the light tip.

The beam profile characterization of the VALO Cordless also showed differences in the irradiance and in the spectral irradiance regardless of the camera used for beam profile analysis. The VALO Cordless is a multiwave LED LCU with three different peaks of emission, two within the blue spectrum (at 440 [cyan-blue] and 460 nm [royal-blue]) and one within the violet spectrum (at 405 nm). The VALO Cordless has a quartz microlens with an external dimension of 1.2 cm in diameter. However, the active area of emission of the VALO Cordless has 0.96 cm in diameter, resulting in an area of emission 0.691 cm ^2^ . The VALO Cordless had an average radiant emittance of approximately 1,066 mW/cm ^2^ . However, for the violet wavelength range, there were areas of higher radiant emittance (~210 mW/cm ^2^ ) on the bottom left part of the light tip, and low radiant emittance (~50 mW/cm ^2^ ) on the top left of the light tip. For the royal-blue wavelength range, there were areas of higher radiant emittance (~1,075 mW/cm ^2^ ) at the top part of the light tip, and low radiant emittance (~106 mW/cm ^2^ ) on the bottom of the light tip. For the royal-blue wavelength range, there were two areas of higher radiant emittance (~2,100 mW/cm ^2^ ), one at the top right and another on the bottom left part of the light tip, and areas of low radiant emittance (~120 mW/cm ^2^ ) on the bottom right of the light tip. These findings are also probably related to the VALO Cordless design. The VALO Cordless has four high-power LED chips emitting different wavelengths, two LED chips emitting royal-blue light, one LED chip emitting cyan-blue light, and one LED chip emitting violet light. All LED chips are located at the center of the light tip associated with a quartz microlens that does mix the light beam throughout the light tip, but not completely.

In summary, a slight difference was noted between cameras, as showed and further explored in the histograms presented in the **Supplementary Material** (available in the online version). The results suggest that the mirrorless camera (NEX-F3) had a somewhat better performance than the smartphone camera (iPhone). However, it is important to point out that both the NEX-F3 and the iPhone cameras data showed a strong correlation with the data obtained using the Ophir camera. Thus, different cameras could be used in dentistry to perform the beam profile analysis of dental LCUs, since minimal requirements for the camera are respected. In a previous study, 5 the authors attempted to use a digital single reflex camera and an iPad camera to analyze the light beam profile of different LCUs qualitatively. However, this study failed to address the statistical analysis of the images, and the conclusions are made on the assumption of visual comparison without any digital image correlation analysis. Besides that, the beam profiles images of the inactive light guide tips do not coincide with the real intensity distribution in all cases. With the referenced study based on imaging comparison in pseudo color, the methods reported do not provide any data reproducibility.

The findings in the present study have significant implications for understanding how imaging sensors’ technology and how to adapt to dental LCU analysis. The methods reported here can contribute tremendously to further research projects and articles that intend to report the light-curing unit spectrum, irradiance, and beam profile. This study demonstrates and guides the reader on making a light beam profiler with different CCD/CMOS sensors. It can provide many authors with the opportunity to report the full characteristic of the LCUs used in their studies inexpensively and reproducibly. As state by Price et al 2 , “for improved interstudy reproducibility, reduced risk of premature failures, and ultimately better patient care, researchers and dentists need to know how to accurately characterize the electromagnetic radiation (light) they are delivering to the resins they are using.” Overall, the present study strengthens the idea that it is very challenging to publish and validate any research paper that does not report the full characteristics of the studies’ LCUs.

Still, a note of caution is due here since the number of LCUs and camera devices evaluated in this study is limited, and a more comprehensive study would include more groups. Questions remain unanswered at present, but this study directs further studies to pursue the development of camera gadgets and software, as well as the validation of these devices according to the ISO standards. Continued efforts are needed to make light beam profile and spectral analysis of the dental LCUs more accessible to dentists in the clinical practice.

## Conclusion

This study demonstrated that mirrorless and smartphone cameras are able to perform beam profiling analysis of dental LCUs. The standard Ophir beam profile system presented the most accurate distribution, but the mirrorless and smartphone cameras presented a strong correlation in the irradiance distribution of the beam profile images. Specific requirements for the camera devices such as type of sensor, image bit depth, and image processing are important to achieve consistent results when using alternative methods for dental LCUs beam profiling.
